# Supplementary material for: Associations of Dietary Zinc–Vitamin B6 Ratio with All-Cause Mortality and Cardiovascular Disease Mortality Based on National Health and Nutrition Examination Survey 1999–2016
Source: Nutrients. 2023 Jan 13;15(2):420. doi: 10.3390/nu15020420 (PMC9864187; doi:10.3390/nu15020420)
Supplement: Supplementary file 1 [file nutrients-15-00420-s001.zip › nutrients-2125463-supplementary.pdf]

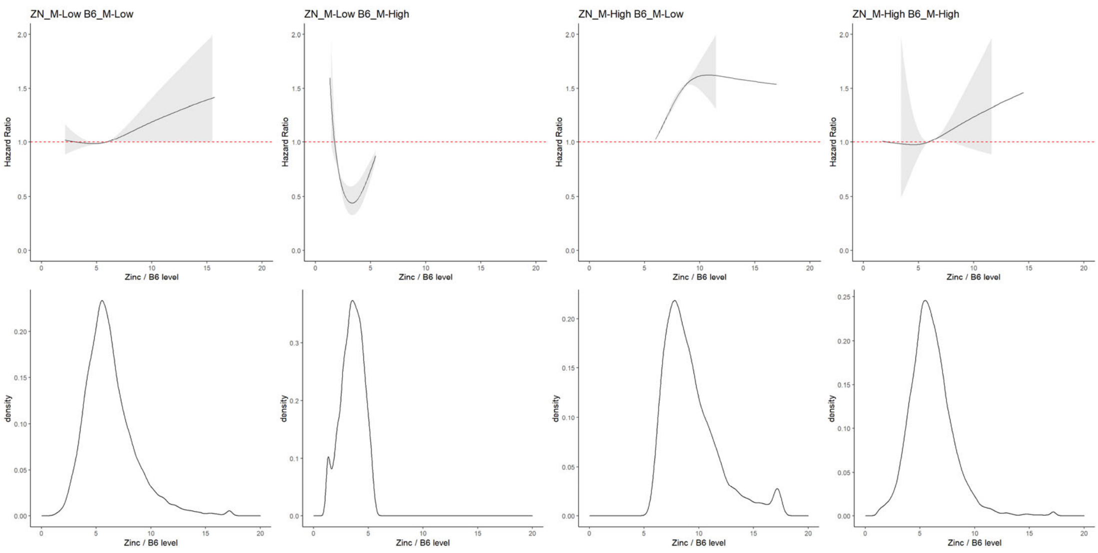

Figure S1. Association of dietary Zinc-vitamin B6 ratio with CVD mortality for different intake groups from NHANES 1999 to 2016
